# Supplementary material for: Self‐Healing Hyaluronic Acid‐based Hydrogel with miRNA140‐5p Loaded MON‐PEI Nanoparticles for Chondrocyte Regeneration: Schiff Base Self‐Assembly Approach
Source: Adv Sci (Weinh). 2024 Nov 5;12(1):2406479. doi: 10.1002/advs.202406479 (PMC11714154; doi:10.1002/advs.202406479)
Supplement: Supplementary file 1 — Supporting Information [file ADVS-12-2406479-s001.docx]

**Self-healing hyaluronic acid based hydrogel with miRNA 140--5p loaded MON-PEI nanoparticles for chondrocyte regeneration: Schiff base Self-Assembly approach**

Wei Zhu^&1^, Han Wang^&1^, Bin Feng^1^, Guangli Liu^1^, Yixin Bian^1^, Tianhao Zhao^1^, Qi Wang^1^, Xisheng Weng*^1^

1. Department of Orthopedics, State Key Laboratory of Complex Severe and Rare Diseases, Peking Union Medical College Hospital, Chinese Academy of Medical Sciences & Peking Union Medical College, 100730 Beijing, China.

*Corresponding author:

Xisheng Weng, email: xshweng@pumch.cams.cn, Tel/Fax: +86 010--69152709

^&^These authors contributed equally to this work.

**Image deleted. Please check.**

**Schematic illustrations of Self-healing hyaluronic acid based hydrogel with miRNA 140--5p loaded MON-PEI nanoparticles for chondrocyte regeneration.** The self-healing hydrogel was prepared under UV cross-linking with mineralized silk fibroin and miR-140-5p-loaded MON-PEI nanoparticles incorporated. Then it was used for chondrocyte regeneration in rabbit cartilage defect and achieved better effect.

**Abstract**

Articular cartilage defects present a significant therapeutic challenge due to the inherent avascular and aneural characteristics of cartilage tissue. Gene therapy has emerged as a promising strategy for cartilage regeneration, particularly through the use of functional RNA and biomaterial-assisted frameworks. In this study, we developed and fabricated an innovative gene-activated self-healing hydrogel for the controlled release of miR-140-5p, a key regulator of cartilage regeneration. The hydrogel, crosslinked via UV radiation, is composed of aminated hyaluronic acid and a modified photosensitizer (NB). To enhance the scaffold’s structural integrity and gene delivery efficiency, mineralized silk fibroin and miR-140-5p-loaded MON-PEI nanoparticles were incorporated. Our findings demonstrate that this novel hydrogel (miR140-5p-CaP@mSF-HA-NB) effectively encapsulates and releases miR-140-5p, exhibits excellent biocompatibility, and promotes enhanced cartilage regeneration in both in vitro and in vivo models. Therefore, this gene-activated hydrogel holds significant potential for clinical applications in the treatment of articular cartilage defects.

**Key Words**:Self-healing，miRNA 140--5p，hydrogel，chondrocyte regeneration

**1.** **Introduction**

Articular cartilage, which has limited self-healing capacity, is predominantly located at the extremities of mobile bones^1^. The degeneration of articular cartilage can lead to the onset of osteoarthritis (OA), a disease associated with significant clinical and socioeconomic burdens^2,3^. Small chondral defects, frequently observed in OA patients, are critical contributors to disease progression^4^. The targeted repopulation of mesenchymal stem cells (MSCs) with chondrogenic potential into these lesions is a key approach in cartilage tissue engineering, currently considered one of the most effective therapeutic strategies^5,6^. However, stem cell-based therapies face limitations, including reduced mechanical strength and the relatively weak capacity of MSCs to induce cartilage formation in a directed manner.

Due to their precise spatial and temporal regulation of target genes and associated pathways, microRNAs are implicated in pivotal roles in cartilage development and chondrogenesis^7^. Notably, diminished levels of miR140-5p have been identified in progenitor/stem cells (CPCs) of osteoarthritis (OA) cartilage, correlating with OA progression^8^. Currently, there is a burgeoning interest in investigating efficient gene vector delivery systems as a prominent focus in advancing gene therapy methodologies^9, 10^

Biomaterials have recently garnered increased attention as promising therapeutic options for advancing cartilage repair processes^11^. This exploration encompasses controlled release of therapeutic factors^12^, protein binding mechanisms^13^, and localized gene vector delivery^14^. Biomaterials employed in cartilage regeneration primarily fall into categories such as ceramic-based formulations, synthetic or natural polymer formulations, and hybrid materials^15^. Notably, hydrogels derived from polymer or hybrid materials exhibit commendable biocompatibility and injectability. These hydrogels not only enhance lubrication but also provide a suitable 3D extracellular environment (ECM), rendering them conducive to tissue regeneration and the treatment of localized cartilage lesions^16, 17^. The application of natural or biomimetic polymers for the synthesis of hydrogels allows the preparation of scaffolds with properties close to the native ECM, including hyaluronic acid, collagen, gelatin, chitosan, chondroitin sulfate and silk fibrin. Silk fibroin (SF), a natural protein fiber FDA-approved for certain biomedical devices such as sutures, has demonstrated remarkable biocompatibility, robust mechanical properties, and adjustable biodegradability^18-20^. SF has been extensively explored and developed in various biomaterial forms, including films, electrospun nanofibers, sponges, and hydrogels, for applications in tissue regeneration^18^. SF hydrogels exhibit a transition from amorphous to intermolecular β-sheet protein conformation, a transformation inducible by factors such as shear stress, organic solvents, ions, low pH, high temperature, and exposure to CO_2_^21-24^. Despite these advantages, the hydrophobic nature of the β-sheet network in silk fibroin contributes to the brittleness of the hydrogels, posing a persistent challenge that requires further exploration and resolution.

On the other hand, the injectable nature of hydrogel scaffolds, capable of conforming to the contours of cartilage defects, is particularly advantageous in preventing complications related to surrounding tissue damage^25^, thereby reducing surgical time and minimizing postoperative scar size^26^. Recent studies have demonstrated that polymeric biomaterials-mediated gene therapy holds considerable promise in the treatment of cartilage injuries. This approach offers dual functionality as gene vector delivery systems and supporting scaffolds, enabling the spatiotemporal regulation of endogenous chondrogenesis^27, 28^. However, designing biomaterials that can effectively carry genes is currently a difficult and hot topic. Pure gene fragments especially micro RNA are sensitive to loss of activity and cannot be effectively transfected into cells to exert their effects. Successfully loading specific gene fragments into self-healing hydrogel and obtaining a stable system may play crucial role in promoting cartilage repair in articular cavity. Meanwhile, self-healing hydrogel can effectively fill the cartilage defect area and then play appropriate mechanical support function.

In this study, we devised a comprehensive approach for the in-situ assembly of a self-healing hydrogel through Schiff base formation under ultraviolet crosslinking, incorporating MON-PEI (Mesoporous organosilicon-polyethyleneimine) nanoparticles for the delivery of miR140-5p. The resultant polymeric hydrogel establishes well-crosslinked 3D polymeric networks, creating an environment akin to the extracellular matrix of native tissues for enhanced cellular support. Concurrently, the encapsulated miR140-5p within nanoparticles can be released, facilitating the promotion of cartilage repair and reconstruction through the modulation of specific pathways. Our biomaterial based on self-healing hydrogel can not only effectively load micro RNA, but also better fill articular cartilage defect area due to its self-healing properties. With hydrogel degradation, the micro RNA loaded in nanoparticles will be released and successfully transfected into cell to play crucial role in cartilage regeneration. The mineralized silk fiber contained in this hydrogel, which further could improve its mechanical properties showing unique advantages and more suitable for joint cavity application. We validated that this self-healing hydrogels could act as an excellent gene therapy delivery system. For the clinical treatment difficulties where the common treatment of cartilage defects can not achieve good repair effect, our biomaterial treatment system provides new ideas and direction for the future treatment.

**2.** **Results and discussion**

2.1 *Characteristic of CaP@mSF and MON-PEI nanoparticles*

According to the infrared spectrum of mSF and CaP@mSF (Figure 1A), the absorption peak at 1650 cm^−1^ of mSF corresponds to the stretching vibration absorption peak of the amide I band C = O. Upon coating calcium phosphate on the surface of mSF, the peak of the amide I band C = O appears at 1634 cm-1, shifting to a lower wavelength, indicating the interaction between Ca2+ and the carboxyl group of microfibers. In the XRD spectrogram (Figure 1B), the broad peak of mSF at 20.7° belongs to the β-folded crystal region, suggesting an orderly internal structure of mSF. After biomineralization for 7 days, the absorption peaks of CaP@mSF appeared at 29.64°, 31.64°, 35.98°, 39.64°, 47.02°, indicating the presence of calcium phosphate on the surface of mSF.

In the thermogravimetric curve (Figure 1C), both mSF and CaP@mSF lost about 8.333% of water mass at around 100°C. After deducting the proportion of water, the residue proportion of mSF was 30.451%, and the residue proportion of CaP@mSF was 37.3%. Therefore, the proportion of calcium phosphate in the prepared CaP@mSF was approximately 6.849%. Micro silk fibers were immersed in mineralizing medium and magnetic agitation was used during mineralization to prevent condensation of mSF. After 7 days of mineralization, calcium phosphate was entirely coated on the mSF sample surface. The scanning electron micrograph of mSF and mineralized samples in simulated body fluid (SBF) buffer solution for 4 and 7 days respectively. As shown in Figure 1D, mSF showed a smooth microfiber structure before mineralization on day 0. The calcium phosphate crystals on surface of mSF appeared with the increase of mineralization time on day 4. After 7 days of mineralization, the mSF surface was almost entirely covered by calcium phosphate.

Figure 1E displays the transmission electron microscope image of the prepared mesoporous organosilicon (MON). The image reveals a snowflake-like porous branching structure in MON, attributed to the inclusion of bis[3-(triethoxysilyl)propyl] tetrasulfide (BTES), enhancing its capabilities for grafting PEI and loading SiRNA. Figures 1H, F, and G illustrate the particle size distribution, average particle size, and Zeta potential of the prepared MON, MON-NH2, MON-COOH, and MON-PEI. During the preparation of MON-COOH, the size of mesoporous silicon particles markedly increased, likely due to the aggregation of carboxyl and amino groups between MON-COOH particles through electrostatic adsorption. After PEI grafting, the particle size of MON-PEI returned to levels similar to MON and MON-NH2, indicating weakened electrostatic interactions between particles resulting from the grafting of carboxyl and PEI in MON-COOH. The final Zeta potential of MON-PEI was 34.1 ± 0.21 mV, providing favorable conditions for SiRNA loading. Mesoporous silica-based nanoparticles (MSN) have emerged as multifunctional platforms with applications in drug delivery, spanning catalysis, nanotechnology and so on^31^. To enhance cell internalization and biodistribution, chemical tailoring to switch from negative to positive surface charge in MSN has been one important strategy. Antonio et al ever reported a series of mesoporous silica nanoparticles including conventional and modified with molecules containing -NH2, -COOH, DIOL or other functional groups MSN. Then it was characterized its Zeta potential distributing from -18mV to +25.5mV, which obviously lower compared with MON-PEI in our study. Another study developed a novel virus-like mesoporous silica nanoparticles with a spiky tubular rough surface via single micelle epitaxial growth approach in a low-concentration-surfactant oil/water biphase system^32^. The structural of parameters of this nanoparticles were characterized with controlled core diameter (60-160nm), tubular length (6-70nm), which is similar in diameter to the MON-PEI nanoparticles and are all larger than the conventional MSN nanoparticles (about 45nm) ^33^. Compared with traditional mesoporous silica-based nanoparticles, the high positive charge of MON-PEI nanoparticles showed advantages on microRNA loading and cell internalization.

Figure 1I presents agarose gel electrophoresis of the MON-PEI/miR-140-5p complex under different N/P ratios to evaluate microRNA loading ability for nanoparticles. Different RNA migration conditions were verified by setting up MON-PEI/miR-140-5p complexes with varying mass ratios. N/P represents the mass ratio of nanoparticles to RNA. For N/P ratios below 15, observable pink bright bands indicated that MON-PEI could not form a stable complex with miR140-5p. However, when the mass ratio exceeded 20, miR140-5p had difficulty migrating from the complex under the electric field, indicating successful wrapping of miR-140-5p by MON-PEI to form a stable complex. This phenomenon may be attributed to the presence of amino groups and positive charges on PEI, facilitating electrostatic interaction with negatively charged genes and rendering it an effective gene delivery carrier. During practical applications, different weight and electric charge are determined by gene sequences length. So when achieving consistent N/P ratios in our system for different genes, it needs to be verified again. Meanwhile, the quantitative collection of genes requires multidisciplinary cooperation.

Image deleted. Please check.

Figure 1. A: Infrared spectrogram of mSF and CaP@mSF. B: X-ray diffraction spectrum of mSF and CaP@mSF. C: Thermogravimetric curve of mSF and CaP@mSF. D: mSF mineralization for 0d, 4d and 7d. E: The TEM of MON nanoparticles. F: The average particle size of MON, MON-MH2, MON-COOH and MON-PEI. G: The zeta potential of MON, MON-MH2, MON-COOH and MON-PEI. H: Particle size distribution diagram of MON, MON-MH2, MON-COOH and MON-PEI. I: Gel electrophoresis of MON-PEI nanoparticle with miR140-5p.

2.2 *Evaluation of biopolymer binders and CaP@mSF-HA-NB hydrogel*

The biopolymer components constituting the hydrogel were characterized through NMR, IR, and UV spectra. In the infrared spectrum (Figure 2A), the absorption peak of HA-NB and AHA around 1650 cm-1 corresponds to the stretching vibration absorption peak of amide I with C = O bond. In the nuclear magnetic resonance spectrum (Figure 2B), HA-NB exhibits two absorption peaks in the range of 78 ppm, attributed to the hydrogen on the benzene ring in the NB structure, indicating successful grafting onto HA. AHA, compared with HA, presents an absorption peak in the range of ppm = 23 due to the hydrogen on the amino group, confirming the success of HA modification. The UV spectrogram (Figure 2C) for HA-NB reveals absorption peaks at 350 nm and 307 nm, indicative of the presence of large π bonds in the NB structure, further supporting successful NB grafting. After UV irradiation, a shift in the absorption wavelength of HA-NB suggests that the structure of O-Nitrobenzyl alcohol in NB undergoes gradual changes under UV irradiation to form an aldehyde group structure. This chemical characteristic enables HA-NB to covalently bond with AHA containing amino groups, forming a hydrogel network. Figure 2E confirms the self-healing ability of this hydrogel, as it demonstrates the hydrogel healing itself after being cut open based on its Schiff base reaction. The green dye was added to this hydrogel, and when the two cut pieces touch again, they will be self healing.

Our hydrogel has self-healing ability, and will gradually spontaneously degrade and release MON-PEI nanoparticles containing miR-140-5p due to natural swelling in the water environment. The release of miR-140-5p into the surrounding environment occurs primarily through simple diffusion, which is the movement of molecules along a concentration gradient, from areas of higher concentration to lower concentration, until uniform distribution is achieved. Additionally, during the degradation of the hydrogel, the collapse of the gel network exposes the MON nanoparticles encapsulated within, thereby accelerating the release of miR-140-5p.

This stability of the fabricated hydrogel was evaluated under lysozyme/PBS solution, and the results were shown in supplementary material Figure 2S. The hydrogels with different concentrations of HA-NB were all degraded gradually with time in lysozyme/PBS solution. The higher the concentration of HA-NB in the hydrogel, the slower its degradation rate. And each hydrogel can be maintained for more than 2 weeks.

Self-healing hydrogels have good self-healing ability, degradability and bio compatibility. However, the lack of mechanical properties and long-term stability in vivo limit their clinical use. In the future, self-healing hydrogels would exhibit better application prospect in the field of medical devices through structural modification to solve the existing problems.

SEM images of freeze-dried hydrogel scaffolds in various groups are depicted in Figure 2D. When the fixed AHA concentration is 2% and the HA-NB concentration increases from 2% to 4%, the size and density of the hydrogel scaffold pores undergo changes. Within this concentration range, elevating the HA-NB concentration enhances the cross-linking degree of the hydrogel network, leading to a gradual reduction in pore size and an increase in pore density. Figure 2F and Figure 2G illustrate the variations in the elastic modulus (G') and viscous modulus (G'') of the UV-cured hydrogel over time and frequency. In all three groups, the hydrogels maintained a state where G' > G'', indicating their stable gel-like form under suitable conditions. As the HA-NB concentration increased, G' exhibited a strengthening trend, signifying an enhancement in the hydrogel’s strength.

Recent years chitosan and nitrogen-doped graphene quantum dots (NGQDs) hydrogels have emerged as promising biomaterial for biomedical application. Livy et al. developed a transparent, elastic, and self heal-able polymer matrix based on NGQDs for wearable healthcare pH sensors on human body^29^. This hydrogel has self-healing ability due to its hydrophilic and hydrophobic structure compared with schiff base in our hydrogel, but it has not performed the function of carrying drugs or genes. Our hydrogels successfully encapsulated micro RNA and effectively transfected into cells. In another study, Darwin et al. reported plasma-engineered smart drug nanocarriers containing NGQDs for drug delivery and pH responsive release^30^. However, this hydrogel did not exhibit self-healing performance and load with conventional anti-tumor drugs, which was different with the microRNA loading in this study hydrogel. Due to the instability of microRNA in common condition, it is important to develop bio materials for micro RNA carrier system. Our hydrogels successfully loaded micro RNA with nanoparticles and transfected into cell to function. On the other hand, NGQDs based hydrogels in these two study showed high toughness, while the primarily hyaluronic acid-based hydrogel in our study was self-healing and lubricative making them more suitable for intra-articular use.

Due to the multiple synthesis process of its component and micro RNA preparation, the manufacturing cost of our hydrogel is high and each synthesis process will lose raw materials. Our unique hydrogel system is currently in the research stage, and the cost would be greatly reduced when fully commercialized or synthesized in the later amount.

Image deleted. Please check.

Figure 2. A: The infrared spectrum of AHA, HA-NB and HA. B: The nuclear magnetic resonance spectrum of AHA, HA-NB and HA. C: The UV spectrogram of AHA, HA-NB and HA. D: The SEM of HA-NB/AHA hydrogel with different ratios of HA-NB. E: The self-healing ability of CaP@mSF-HA-NB hydrogel. F+G: Rheological properties of different groups of hydrogels.

2.3 *miR140-5p-CaP@mSF-HA-NB Behaves High Biocompatibility and Promotes Chondrogenic Differentiation of hBMSCs*

In order to verify CaP@mSF-HA-NB can exhibit good biocompatibility, Cell Counting Kit-8 (CCK-8) were used to detect the proliferation of human bone marrow mesenchymal stem cells (hBMSC) over 5 days when cultured with varying concentrations of material extracts mixed with cell medium, ranging from 0%, 10%, 25%, 50%, to 100%. The culture of hBMSC cells with CaP@mSF-HA-NB extracts at concentrations of 10%, 25%, and 50% significantly increased cell proliferation during the 1--5 day culture period, as shown in Figure 3A. However, at a concentration of 100%, the cell proliferation was not significantly different from that at 0%, indicating excellent biocompatibility of the CaP@mSF-HA-NB.

Previous studies have reported that intra-articularly injected miR-140-5p can efficiently penetrate cartilage and be rapidly internalized by chondrocytes in rat models. The intra-articular administration of miR-140-5p has been shown to improve behavioral scores, increase chondrocyte numbers, enhance cartilage thickness, and ameliorate pathological scores to varying extents. These findings indicate that miR-140-5p exhibits a promising therapeutic effect in the treatment of osteoarthritis (OA)^34^.

Thus we combined the chondrogenesis-promoting miRNA-140-5p with CaP@mSF-HA-NB and validated whether this composite material could exhibit the expected biological functions in vitro and in vivo. Firstly, quantitative real-time PCR was employed to evaluate the efficiency of miR140-5p mimic transfection. The NC group and NC-CaP@mSF-HA-NB showed low miR140-5p expression due to nonsense sequence transfection. The miR140-5p expression in the miR140-5p-CaP@mSF-HA-NB group was significantly higher than that in the miR140-5p group (p < 0.05)(Figure 3B2). This suggested that compared to the group treated with miRNA-140-5p alone, the miR140-5p-CaP@mSF-HA-NB group was more effective in maintaining the stability of miRNA-140-5p.

To further validate the chondrogenic potential of CaP@mSF-HA-NB, hBMSC were treated with NC mimic, miR140-5p mimic, miR140-5p mimic plus CaP@mSF-HA-NB(miR140-5p-CaP@mSF-HA-NB), and Negative Control (NC) mimic plus CaP@mSF-HA-NB (NC-CaP@mSF-HA-NB) for 48 hours, followed by RNA extraction, RT-PCR and transcriptome sequencing(RNA-Seq). RT-PCR was performed to detect the expression of chondrogenic genes, including *ADAMTS5* and *COL2A1*, in the four groups after 48 hours of cell transfection culture. The expression levels of *COL2A1* and *ADAMTS5* in the miR140-5p-CaP@mSF-HA-NB group were significantly higher than those in other groups (*p* < 0.05) (Figure 3B3), suggesting a noticeable tendency towards chondrogenic differentiation in hBMSC following treatment with miR140-5p-CaP@mSF-HA-NB. RNA-Seq results revealed significant differential gene expression among the four groups, with upregulation and downregulation of various genes in the miR140-5p-CaP@mSF-HA-NB vs NC group. KEGG analysis showed enrichment in signaling pathways related to Rheumatoid arthritis, PI3K-Akt, MAPK, TGF-beta, and other pathways associated with arthritis and osteogenesis (Figure 3E). GSEA analysis from DisGeNET also indicated enrichment of DEGs in pathways related to arthritis (Figure 3F). The list of DEGs revealed several genes specifically expressed in chondrocytes, supporting the idea of a pronounced chondrogenic tendency in hBMSC treated with miR140-5p-CaP@mSF-HA-NB (Figure 3G).

Image deleted. Please check.

Figure3: miR140-5p-CaP@mSF-HA-NB exhibited excellent biocompatibility and facilitated chondrogenic differentiation of hBMSCs. A.The CCK-8 results depict the proliferation of hBMSCs over 5 days when cultured with varying concentrations of material extracts mixed with cell medium, ranging from 0%, 10%, 25%, 50%, to 100%. B. The expression of miR140-5p, *ADAMTS5* and *COL2A1* in NC, miR140-5p, NC-CaP@mSF-HA-NB and miR140-5p-CaP@mSF-HA-NB four groups after 48 hours cell transfection culture(**p* < 0.05, ***p* < 0.01, ****p* < 0.005, *****p* < 0.001). C. RNA-Seq heatmap showing the DEGs in NC, miR140-5p, NC-CaP@mSF-HA-NB and miR140-5p-CaP@mSF-HA-NB four groups((n = 3 for each group). D.Scatter plot of transcriptome data in miR140-5p-CaP@mSF-HA-NB vs NC(p < 0.05, |log 2 (fold change)| > 0). E. KEGG classification of DEGs in miR140-5p-CaP@mSF-HA-NB vs NC. F. GSEA results of DisGeNET enrichment analysis in miR140-5p-CaP@mSF-HA-NB vs NC. G. DEGs list of Osteoarthritis_knee(C0409959, DisGeNET) in miR140-5p-CaP@mSF-HA-NB vs NC.

2.4 *MiR140-5p-CaP@mSF-HA-NB Treatment Promotes Chondrocyte Proliferation in OA cell model*

To further assess the therapeutic effects of miR140-5p-CaP@mSF-HA-NB on arthritis, an arthritis model was induced using IL-1β with the human chondrocyte cell line C28/I2. In vitro validation was conducted to evaluate cell proliferation using EdU staining to visually assess and quantify the proliferation activity of C28/I2 cells pre- and post-arthritis induction, when treated with CaP@mSF-HA-NB, NC-CaP@mSF-HA-NB and miR140-5p-CaP@mSF-HA-NB in the arthritic cell model.

The previous studies have reported that: in late-stage OA, the cartilage becomes hypocellular, often accompanied by lacunar emptying, which has been considered as evidence that chondrocyte death is a central feature in OA progression. Apoptosis clearly occurs in osteoarthritic cartilage^35^.The effect of miR-140-5p on OA Cartilage progenitor/stem cells (CPCs) fate reprogramming and the potential mechanisms were validated in OA rats. Reduced miR-140-5p were observed in OA CPCs and associated with OA progression. IL-1β induced OA-like changes in CPCs fate, which could be exacerbated by miR-140-5p inhibitor while alleviated by DAPT (a specific Notch inhibitor) and miR-140-5p mimic. MiR-140-5p protects CPCs from fate changes via inhibiting Jagged1/Notch signaling in knee OA, providing attractive targets for OA therapeutics^36^.

In comparison to the control group, the proliferation of C28/I2 cells in the IL-1β-induced arthritis model was notably suppressed. However, treatment with miR140-5p-CaP@mSF-HA-NB restored the proliferation rate to normal levels (Figure 4A and 4B). Additionally, cell apoptosis was evaluated via flow cytometry. The proportions of apoptotic cells in different groups were observed, and it was found that compared to the control, cell apoptosis significantly increased in the IL-1β group. Treatment with miR140-5p-CaP@mSF-HA-NB completely reversed this effect (Figure 4C and 4D). These findings from flow cytometry were consistent with those obtained from the EdU assay, collectively indicating that miR140-5p-CaP@mSF-HA-NB facilitated cell proliferation and attenuated cell apoptosis in osteoarthritis (OA) cells.

Furthermore, cell cycle analysis by flow cytometry revealed the distribution of C28/I2 cells in different phases. The percentage of G1 phase cells in the IL-1β group was significantly higher than that in the control group. Cells in the IL-1β group exhibited a propensity to arrest in the G1 phase, with a marked decrease in S phase cells, whereas treatment with miR140-5p-CaP@mSF-HA-NB reversed the IL-1β-induced alterations in the cell cycle (Figure 4E and 4F). Since progression from the G1 phase to the S phase is critical for cell proliferation and cellular aging is characterized by a decline in replicative ability. These may suggest that chondrocytes in the OA model underwent aging, leading to slower RNA and protein biosynthesis and reduced cell proliferation. The miR140-5p-CaP@mSF-HA-NB treatment appeared to reverse the aging of C28/I2 cells in the OA model, thereby restoring their proliferative capacity.

Overall, results of Figure4 indicate that miR140-5p-CaP@mSF-HA-NB treatment has positive effects on cell proliferation and apoptosis in an OA cellular model.

Image deleted. Please check.

Figure4: miR140-5p-CaP@mSF-HA-NB reversed the negative effect of IL-1β on chondrocyte proliferation in osteoarthritis. C28/I2 cells were divided into five groups: blank, IL-1β, IL-1β+CaP@mSF-HA-NB, IL-1β+miR140-5p-CaP@mSF-HA-NB, and IL-1β +NC- CaP@mSF-HA-NB. A. EdU staining for evaluation of the influences of five groups on the proliferation of C28/I2. The new generation cells were detected via EdU (green). DAPI stained nuclei in blue. Merged view of EdU (green) and DAPI (blue) showing the overlap. B.Statistical results of proliferating cells in EdU staining. C. Flow cytometry results depicting cell apoptosis in C28/I2 cells across five experimental groups. D. Statistical results of apoptosis cells in flow cytometry. E. Flow cytometry analysis depicting the cell cycle distribution in C28/I2 cells among five experimental groups. F.Statistical results of cell cycle distribution in flow cytometry. (**p* < 0.05, ***p* < 0.01, ****p* < 0.005,*****p* < 0.001).Each experiment was performed in duplicate at least 3 times.

2.5 *MiR140-5p-CaP@mSF-HA-NB Treatment alleviate Chondrocyte inflammation in OA cell model*

To characterize the impact of miR140-5p-CaP@mSF-HA-NB on inflammatory cytokine levels, concentrations of IL-6, TNF-α, and NO in the supernatant of cell cultures were assessed using ELISA. The findings revealed significantly higher levels of IL-6, TNF-α, and NO in the IL-1β group compared to the blank group. Furthermore, the miR140-5p-CaP@mSF-HA-NB group exhibited significantly decreased levels of IL-6, TNF-α, and NO compared to the IL-1β group (Fig. 5A). These results collectively suggest that miR140-5p-CaP@mSF-HA-NB effectively reduces the concentrations of IL-6, TNF-α, and NO.

Excessive ROS can induce apoptosis of chondrocytes, activate the production of inflammatory factors, and lead to the degradation of articular cartilage. Flow cytometry analysis was performed to quantify ROS levels across various experimental groups, including blank, IL-1β, IL-1β + CaP@mSF-HA-NB, IL-1β + miR140-5p-CaP@mSF-HA-NB, and IL-1β + NC-CaP@mSF-HA-NB, yielding percentages of 1.27%, 7.64%, 4.61%, 1.73%, and 4.60%, respectively. The results revealed that treatment with miR140-5p-CaP@mSF-HA-NB reduced ROS levels in OA cells to levels approximating those of non-OA cells, indicating the lowest oxidative damage and significant alleviation of the inflammatory response in this group (Figure 6B and 6C).

Image deleted. Please check.

Figure5: MiR140-5p-CaP@mSF-HA-NB treatment alleviate chondrocyte inflammation in OA cell model. C28/I2 cells were divided into five groups: blank, IL-1β, IL-1β + CaP@mSF-HA-NB, IL-1β + miR140-5p-CaP@mSF-HA-NB, and IL-1β + NC-CaP@mSF-HA-NB. A. ELISA results of IL6, NO and TNFα in C28/I2 cells across five experimental groups. B.Flow cytometry results depicting ROS level in C28/I2 cells across five experimental groups. C. Statistical results of ROS level in flow cytometry. (*p < 0.05, **p < 0.01, ***p < 0.005,****p < 0.001).Each experiment was performed in duplicate at least 3 times.

2.6 *MiR140-5p-CaP@mSF-HA-NB for In Vivo Treatment of Rat Tibial Articular Cartilage Defects*

In vivo, femoral condyle cartilage defects were induced in rats using an intraosseous approach, followed by treatment with blank(Figure6A), NC-CaP@mSF-HA-NB(Figure6B) and miR140-5p-CaP@mSF-HA-NB(Figure6C) at the defect site. After an 8-week period, samples were collected for microCT imaging(Figure6A1,A2,B1,B2,C1 and C2) and Safranin O-Fast Green staining(Figure6A4,A5,B4,B5,C4 and C5). The microCT images and gross photographs revealed effective repair of the femoral condyle cartilage defects in the group treated with miR140-5p-CaP@mSF-HA-NB. Safranin O-Fast Green staining provides a visual representation of the structure of articular cartilage and subchondral bone. Basophilic cartilage binds to the basic dye Safranin O, appearing red, while acidophilic bone binds to the acidic dye Fast Green, appearing blue. Consistent with the image below, the red areas representing cartilage progressively increase from left to right, indicating that the miR140-5p-CaP@mSF-HA-NB effectively promotes cartilage repair.

Image deleted. Please check.

Figure 6: A1 and A2: Micro-CT scans of the femoral condyle cartilage defects in the blank group rats; B1 and B2: Micro-CT scans of the femoral condyle cartilage defects in the NC-CaP@mSF-HA-NB group rats; C1 and C2: Micro-CT scans of the femoral condyle cartilage defects in the miR140-5p-CaP@mSF-HA-NB group rats; A3: Gross morphology of the femoral condyle defects in the blank group rats; B3: Gross morphology of the femoral condyle defects in the NC-CaP@mSF-HA-NB group rats; C3: Gross morphology of the femoral condyle defects in the miR140-5p-CaP@mSF-HA-NB group rats. A1 and A2: Safranin O-Fast Green staining of the femoral condyle cartilage defects in the blank group rats; B1 and B2: Safranin O-Fast Green staining of the femoral condyle cartilage defects in the NC-CaP@mSF-HA-NB group rats; C1 and C2: Safranin O-Fast Green staining of the femoral condyle cartilage defects in the miR140-5p-CaP@mSF-HA-NB group rats.n = 3.

**3.** **Conclusion**

In this study, our biomaterial has been proven to effectively loading and delivery system for microRNA-140-5p, and validated at the cellular, molecular, and animal levels in promoting chondrogenesis.The self-healing properties of hydrogels are provided by the dynamic Schiff base bond between HA-NB and AHA. After damaged by external force, the broken part of this hydrogel will be bonded again as a whole through the formation of the Schiff base bond when contacting again. This can avoid the breakage of hydrogel in vivo application. Meanwhile, self healing hydrogel based on hyaluronic acid is more suitable for intra-articular use because of its lubricating property. Chondrogenesis is a big clinical challenge, and our biomaterials provide new ideas and hope for solving it.

**4.** **Experimental section**

4.1 *Preparation of mSF Coated with CaP Particles*

10 g cut pieces silk cocoons were boiled in 1L of 0.5wt% Na_2_CO_3_ solution for 30 minutes, then rinse thoroughly with distilled water to degum the silk fibroin protein. This degumming process was repeated twice and then washed clean and dried. The completely degummed silk fibroin was soaked in 1 M sodium hydroxide solution at 40°C for 5 h to hydrolyze macro silk fibers to prepare microfibers. The alkaline hydrolysis is stopped by centrifugal removal of the lye and then washed several times with distilled water to remove the remaining NaOH. Micro silk fibers (mSF) would be acquired after drying in a blast oven. To obtain in situ mineralization of mSF, 1 g of mSF was immersed in 250mL sterile 1.5 x SBF (mineralizing medium). Magnetic agitation was used during mineralization to prevent condensation of mSF, and the mineralized medium is renewed once a day. After 7 days of mineralization, CaP coated mSF (CaP@mSF) was obtained by repeated centrifugation and washing with water.

4.2 *Preparation of MON-PEI nanoparticles*

First, 2 g cetyltrimethylammonium chloride (CTAC) and 0.1 g triethanolamine (TEA) were dissolved in 20 ml distilled water at 95°C with stirring for 1 h. Then 1.5 mL ethyl orthosilicate (TEOS) and 1.3g bis [3-(triethoxysilyl) propyl] tetrasulfide (BTES) were added with stirring for 1 h. The precipitation was obtained by centrifugation at 15,000 rpm for 15 min and washed with ethanol 3 times to remove the residual reactants to achieve MON. The MON was dispersed in 50 ml ethanol and 200 μl of APTES was added to the solution. The solution was reflowed at 60°C for 4 h. After centrifugation and water washing, amino-functionalized mesoporous silicon (MON-NH_2_) was obtained and re-dispersed in ethanol. Dissolve 30 mg succinic acid, 39 mg EDC and 39 mg HOBT in 5 mL ethanol. Then, a solution containing 30 mg MON-NH_2_ was added to the above solution. After stirring for 5h, carboxy-functionalized MON (Mon-COOH) was centrifuged, washed repeatedly with ethanol and water to remove excess succinic acid, EDC and HOBT, and dispersed in ethanol. After that, 40 mg EDC and 40 mg HOBT were dissolved in 30 mg MON-COOH dispersion solution and treated with ultrasound for 30 min. Then, carboxy-activated MON-COOH dispersed droplets were added to an aqueous solution containing 20 mg PEI. Then, stirred for 5h. MON-PEI was obtained after centrifugation and washing repeatedly with ethanol and water.

4.3 *Preparation of CaP@mSF Modified HA-NB hydrogel* *Synthesis of AHA and HA-NB*

4-(4-(hydroxymethyl)-2-methoxy-5-nitrophenoxy) butyrylethylenediamine (NB) was synthesized according to previously reported procedure^29^ and the chemical structural formula was shown in the supporting material. Weigh 1g hyaluronic acid (HA) and dissolved in 50 mL deionized water. 0.5 g EDC was added in above solution and stirred for 15 min. Then added 0.4 g NHS and waited for 15 min reaction to acquire the AHA solution. 0.5 g NB was added into the above solution for overnight reaction and put the solution into dialysis bag (MWCO = 3500). HA-NB was obtained by freeze-drying in deionized water for 2 days under the condition of avoiding light.

4.4 *Preparation of CaP@mSF Modified HA-NB hydrogel*

First, we prepared 0.05g/ml HA-NB and AHA solution. CaP@mSF was dispersed into HA-NB solution at 0.01g/ml. Then, mixed HA-NB and AHA solution in 1:1 volume, irradiated with 356 nm ultraviolet light for 3~5 minutes, and standed for a period of time to form CaP@mSF Modified HA-NB hydrogel.

4.5 *Characterization of the bio-materials*

mSF and CaP@mSF were inspected by X-ray diffraction (XRD-7000, Shimadzu), SEM (Hitachi S4800, Hitachi High-Technologies company), infrared spectrum (TENSOR 27, Brooke, Germany) and thermogravimetric analysis (STA 449F3, NETZSCH). The biopolymer binders of HA, AHA, HA-NB was characterized by nuclear magnetic resonance spectroscopy (Inova-500M, Varian, USA), infrared spectrum and ultraviolet spectrum (UV1800, Shimadzu). The nanoparticles of MON, MON-NH_2_, MON-COOH, MON-PEITEM were examined by TEM (Hitachi, Japan) and DLS analysis (Zeta-Sizer Nano ZS, Marvin, UK).

The microstructure of synthesized CaP@mSF Modified HA-NB hydrogel was observed by SEM and TEM. Rheometer (Kinexus, Marvin, UK) was used for rheological measurement. G ‘represents the elastic modulus of the sample, and G "represents the viscous modulus of the sample. Conduct dynamic strain scanning at room temperature from 0.1 to 10 rad/s, determine the linear viscoelastic range of the hydrogel, and record the change curve of storage modulus (G’) and loss modulus (G ‘’).

4.6 *Gel electrophoresis*

The gene/polymer complexes with different N/P ratios (N/P ratios: 0:1, 1:1, 2:1, 5:1, 10:1, 15:1, 20:1, 25:1, 30:1, 40:1) were prepared and incubated at room temperature for 30min to obtain the composite solution of materials/SiRNA with different N/P ratios. Used a micro pipette gun to add the sample into the gel sample prepared in advance, and started electrophoresis. The electrophoresis condition was constant voltage 140V, and the electrophoresis time was 30min. Then used a dark box fluorescent detector to show fluorescence, observed and took photos for analysis.

4.7 *hBMSC proliferation and detection of cytocompatibility*

HBMSC were cultured using complete cell culture medium (Cyagen, HUXMA-90011, China) containing 10% (w/v) fetal bovine serum (FBS) in 5% CO_2_ air containing under 37 °C. 10ml medium and 1g CaP@mSF-HA-NB hydrogel was mixed for 24h in 5% CO_2_ air containing under 37 °C and biomaterial release medium was collected and filtered with 0.22um mesh (Merck Millipore, USA). Initially, 2000 cells were seeded in 96-well plate for 12h to allow the cells fully adhering to the wells. Mixed biomaterial release medium with the cell culture medium and set the concentration gradient as 0%, 10%, 25%, 50% and 100%. Added the mixed culture medium into the 96-well plate, and cell counting kit (CCK-8, BOSTER China) was used to measure the active number of these cells on the 1st, 3rd and 5th day respectively.

4.8 *C28/I2 Cell Culture and Induction of Osteoarthritis Chondrocytes by IL-1β*

The Human Chondrocyte Cell Line C28/I2 (Merck, #SCC043, USA) was cultured in DMEM medium (Gibco, Grand Island, NY, USA) supplemented with 0.1 mg/mL G-418 (Gibco) and 10% fetal bovine serum (Gibco). The cells were maintained in a humidified atmosphere with 5% CO_2_ at 37°C. IL-1β (Sigma-Aldrich, St. Louis, MO, USA) was dissolved in double-distilled water following the manufacturer’s instructions, with a storage concentration of 5 mg/mL. Subsequently, IL-1β was diluted to 1 µg/mL using serum-free DMEM/F12 medium. C28/I2 cells were treated with 10 ng/mL IL-1β for 1 hour after being plated onto six-well plates and cultured for 24 hours to induce a cellular osteoarthritis model.

4.9 *RNA extraction and quantitative real time-PCR*

In cell experiment, we divided into four groups. NC group referred to the culture of cells in DMEM basic medium and transfection of nonsense sequence into hBMSC following the lipo3000 transfection protocol (invitrogen，USA). The miR140-5p group referred to transfection of miR140-5p mimics (from Guangzhou Ribobio) into hBMSC using liposomes alone. NC-CaP@mSF-HA-NB group referred to the culture of hBMSC in complicated hydrogel with MON-PEI nanoparticles loaded with nonsense sequence. miR140-5p-CaP@mSF-HA-NB group referred to the culture of hBMSCs with hydrogel extract of nanoparticle complex miR140-5p mimics. 1.2× 10^6^ hBMSC cells were seeded in 6-well plates 18--24h prior to transfection. Media were replaced with antibiotics-free media 6--12 h before transfection. Transfection cells were cultured for an additional 48 h and then washed twice with PBS and lysed in TRIzol reagent (Life Technology, #15596,USA) for total RNA extraction. miRNA was quantified by synthesizing cDNA using a Sangon Biotech miRNA First Strand cDNA synthesis (tailing reaction) kit (B532451), in accordance with the manufacturer’s protocol then performing Quantitative real-time PCR using a microRNA qPCR kit with SYBR Green(Takara Bio Inc) in ABI PRISM 7500 Sequence Detection System (Applied Biosystems, Foster City, CA) using the following thermocycling conditions: pre-denaturation at 95 °C for 30 s, followed by 40 cycles of denaturation at 95 °C, 5 s; annealing at 60 °C, 30 s; and extension at 72 °C, 30 s. The relative expression of each gene was calculated using the 2−ΔΔCT method after normalization to U6 expression.

Total RNA from hBMSC was extracted using Trizol reagent ((Invitrogen, California, USA)). A One Step SYBR® PrimeScript™ qPCR kit (TaKaRa Bio, Otsu, Japan) was used to synthesize cDNA, in accordance with the manufacturer’s instructions. Quantitative real-time PCR (qPCR) was performed using SYBR® Premix Ex Taq™ (TaKaRa) in a Bio-Rad CFX96™ real-time PCR system using the following thermocycling conditions: pre-denaturation at 95 °C for 5s, followed by 40 cycles of denaturation at 95 °C, 10 s; annealing at 57 °C, 20s; and extension at 72 °C, 20s. The relative expression of the specified genes was calculated using the 2−ΔΔCT method after normalization to GAPDH expression. The primers of real time PCR were showed in **Table 1**.

| **Genes** | **Sequence (5'-3') sense** |
| --- | --- |
| *GAPDH*-hum-F2 | 5'-ggAgCgAgATCCCTCCAAAAT-3' |
| *GAPDH*-hum-R2 | 5'-ggCTgTTgTCATACTTCTCATgg-3' |
| *ADAMTS5*-hum-F | CCTGGTCCAAATGCACTTCAGC |
| *ADAMTS5*-hum-R | TCGTAGGTCTGTCCTGGGAGTT |
| *COL2A1*-hum-F | CCTGGCAAAGATGGTGAGACAG |
| *COL2A1*-hum-R | CCTGGTTTTCCACCTTCACCTG |
| Hsa-miR140-5p | CAGUGGUUUUACCCUAUGGUAG |

Table 1. Primers used for real-time PCR.

4.10 *Detection of cell proliferation using EdU assay*

Forty-eight hours post-transfection, cells were seeded into 24-well plates at a density of 3 × 10⁴ cells per well. Cell proliferation was assessed using the EdU incorporation assay. Briefly, cells were incubated with 10 μM EdU, as part of the BeyoClick™ EdU Cell Proliferation Kit with Alexa Fluor 488 (C0071S, Beyotime, China), for 12 hours. Following incubation, cells were fixed in 4% paraformaldehyde for 15 minutes at room temperature and washed three times with phosphate-buffered saline (PBS). The cells were then subjected to click chemistry-mediated conjugation with Alexa Fluor 488 using the provided anti-EdU antibody according to the manufacturer’s protocol. Nuclear counterstaining was performed using 4′,6-diamidino-2-phenylindole (DAPI) solution. EdU-positive cells (green fluorescence) and total nuclei (blue fluorescence) were visualized using a fluorescence microscope, and the percentage of proliferating cells was quantified using ImageJ 1.52 software (National Institutes of Health, Bethesda, MD, USA).

4.11 *Detection of cell apoptosis, cell cycle and ROS by flow cytometry*

C28/I2 chondrocyte cells were pretreated with 10 ng/mL IL-1β (PeproTech, USA) for 1 hour to induce an inflammatory response. Following pretreatment, the cells were exposed to different test materials for an additional 48 hours. Apoptosis was then evaluated using an Annexin V-FITC/Propidium Iodide (PI) Apoptosis Detection Kit (A005-3, 7sea Biotech, China) in accordance with the manufacturer’s instructions. Briefly, cells were stained with Annexin V conjugated to fluorescein isothiocyanate (FITC) and propidium iodide (PI), washed twice with cold PBS, and fixed in 4% paraformaldehyde. The stained cells were subsequently analyzed using a BD Accuri C6 flow cytometer (BD Biosciences, Franklin Lakes, NJ, USA), and data were processed using FlowJo software (Tree Star Inc., Ashland, OR, USA). Meanwhile, the cells were detached from the culture surface and harvested by centrifugation. They were then resuspended in pre-chilled 75% ethanol and fixed at 4°C overnight. Following fixation, the cells were washed and centrifuged, then resuspended in phosphate-buffered saline (PBS) at a volume of 450 µL per sample. Propidium iodide (PI, 50 µL of 0.5 mg/mL solution) was added to each sample for DNA staining. The cell suspensions were incubated at 37°C in the dark for 30 minutes. After incubation, the cells were resuspended in PBS, and the cell cycle distribution was analyzed using flow cytometry (FACSCalibur, Becton, Dickinson and Company, NJ, USA). The analysis was performed using a Cell Cycle Assay Kit (C001-50, 7sea biotech, China). ROS was assessed using Reactive Oxygen Species Assay Kit（S0033S, Beyotime, China）according to the manufacturer’s instructions.

4.12 *Quantification of Inflammatory Cytokines*
To investigate the role of inflammatory cytokines in osteoarthritis (OA) progression, levels of interleukin-6 (IL-6), tumor necrosis factor-alpha (TNF-α), and nitric oxide (NO) were measured in the culture supernatants of IL-1β-pretreated C28/I2 chondrocytes following transfection. Cytokine concentrations were quantified using enzyme-linked immunosorbent assay (ELISA). Specifically, IL-6 was measured with a human IL-6 ELISA kit (ab178013, Abcam), TNF-α with a TNF-α ELISA kit (ab181421, Abcam), and NO with a nitric oxide ELISA kit (RAB0308, Sigma-Aldrich). Each assay was performed in triplicate. Optical density (OD) values were recorded using a multifunctional microplate reader (Varioskan Flash, Thermo Fisher Scientific, Waltham, MA, USA).

4.13 *Transcriptome sequencing*

HBMSCs from the four experimental groups were cultured for 48 hours to induce chondrogenic differentiation. Post-cultivation, cells were detached using 0.25% trypsin-EDTA and collected by centrifugation. The cell pellets were resuspended in phosphate-buffered saline (PBS), and supernatants were discarded. This process was repeated for a total of three replicates per group. Total RNA was extracted from each sample using the TRIzol reagent (Ambion Life Technologies, Carlsbad, CA, USA) following the manufacturer’s protocol. RNA quantity and quality were assessed using the RNA Nano 6000 Assay Kit on the Bioanalyzer 2100 system (Agilent Technologies, CA, USA). RNA sequencing (RNA-seq) libraries were prepared and sequenced on an Illumina HiSeq 2000/2500 platform (Illumina, San Diego, CA, USA). The resulting single-end clean reads were mapped to the reference genome using TopHat v2.0.9 (Johns Hopkins University, Baltimore, MD, USA). Differential gene expression between groups was analyzed using the DESeq2 R package (v1.10.1, Bioconductor). Genes with adjusted p-values (Padj) < 0.05 were considered significantly differentially expressed.

*4.14 Establishment of rabbits model for articular cartilage defects*

Our study was approved by the ethical committee of Peking Union Medical College Hospital(XHDW-2023-116). Healthy male New Zealand white rabbits aged 6--7 months, weighing 2.5-3 kg, were selected for the study. Routine anesthesia was administered, and after shaving and disinfection, the animals were placed in the supine position and draped with a sterile surgical towel. A medial parapatellar incision was made, and the skin and subcutaneous tissue were dissected layer by layer to expose the joint cavity. The patella was everted, and the knee was flexed to expose the femoral condyle. Using a low-speed drill, an articular cartilage defect with a diameter of 5 mm and a depth of 6 mm was created in the weight-bearing region of the femoral condyle, thereby establishing the model. For defect repair, NC-CaP@mSF-HA-NB and miR140-5p-CaP@mSF-HA-NB materials with a diameter of 5 mm and a depth of 6 mm was implanted into the defect area. In the control group, no graft was implanted in the cartilage defect area. The patella was repositioned, and the incision was closed layer by layer without the need for fixation, allowing free movement. The animals were housed in individual cages under the same conditions and sacrificed for specimen collection after 8 weeks. The repair status of the cartilage defect surface was assessed through gross observation, micro-CT, and histological sections, followed by Safranin O-Fast Green staining. The detailed experimental procedure for Safranin O-Fast Green staining is as follows: Slides were deparaffinized and rehydrated to distilled water. Sections were stained with Weigert’s iron hematoxylin working solution for 10 minutes, followed by washing under running tap water for 10 minutes. Fast green FCF solution was applied for 5 minutes, after which the sections were briefly rinsed with 1% acetic acid solution for 10--15 seconds. The sections were then stained with 0.1% safranin O solution for 5 minutes. Dehydration and clearing were performed using sequential treatments of 95% ethanol, absolute ethanol, and xylene, with two changes of each, for 2 minutes per change. Finally, the slides were mounted with a resinous medium.

**Acknowledgments:** This study was supported by grants from National Natural Science Foundation Youth Fund (Grant NO. 82102582)；Natural Science Foundation of Beijing, China(Grant NO.7232119)

**Contribution:** Wei Zhu conducted the materials characterization and drafted the manuscript; Han Wang performed the majority of the cellular experiments and contributed significantly to the writing of the manuscript; Guangli liu and Tianhao Zhao conduted part of the cellular experiments and animal experiments; Yixin Bian contributed to data analysis; Qi Wang contributed to manuscript writing; Xisheng Weng conceived and supervised the experiments.

1. Kwon, H.; Brown, W. E.; Lee, C. A.; Wang, D.; Paschos, N.; Hu, J. C.; Athanasiou, K. A., Surgical and tissue engineering strategies for articular cartilage and meniscus repair. *Nature reviews. Rheumatology* **2019,** *15* (9), 550--570.

2. Rim, Y. A.; Nam, Y.; Ju, J. H., The Role of Chondrocyte Hypertrophy and Senescence in Osteoarthritis Initiation and Progression. *International journal of molecular sciences* **2020,** *21* (7).

3. Berenbaum, F.; Wallace, I. J.; Lieberman, D. E.; Felson, D. T., Modern-day environmental factors in the pathogenesis of osteoarthritis. *Nature reviews. Rheumatology* **2018,** *14* (11), 674--681.

4. Madry, H.; Kon, E.; Condello, V.; Peretti, G. M.; Steinwachs, M.; Seil, R.; Berruto, M.; Engebretsen, L.; Filardo, G.; Angele, P., Early osteoarthritis of the knee. *Knee surgery, sports traumatology, arthroscopy : official journal of the ESSKA* **2016,** *24* (6), 1753--62.

5. De Bari, C.; Roelofs, A. J., Stem cell-based therapeutic strategies for cartilage defects and osteoarthritis. *Curr Opin Pharmacol* **2018,** *40*, 74--80.

6. Arshi, A.; Petrigliano, F. A.; Williams, R. J.; Jones, K. J., Stem Cell Treatment for Knee Articular Cartilage Defects and Osteoarthritis. *Current reviews in musculoskeletal medicine* **2020,** *13* (1), 20--27.

7. Lu, H.; Wei, J.; Liu, K.; Li, Z.; Xu, T.; Yang, D.; Gao, Q.; Xiang, H.; Li, G.; Chen, Y., Radical-Scavenging and Subchondral Bone-Regenerating Nanomedicine for Osteoarthritis Treatment. *ACS nano* **2023,** *17* (6), 6131--6146.

8. Chen, Y.; Huang, H.; Zhong, W.; Li, L.; Lu, Y.; Si, H. B., miR-140-5p protects cartilage progenitor/stem cells from fate changes in knee osteoarthritis. *Int Immunopharmacol* **2023,** *114*, 109576.

9. Song, X.; Liu, C.; Wang, N.; Huang, H.; He, S.; Gong, C.; Wei, Y., Delivery of CRISPR/Cas systems for cancer gene therapy and immunotherapy. *Advanced drug delivery reviews* **2021,** *168*, 158--180.

10. Nagelli, C. V.; Evans, C. H.; De La Vega, R. E., Viral Gene Delivery in Chondrocytes. *Methods in molecular biology (Clifton, N.J.)* **2023,** *2598*, 289--300.

11. Cucchiarini, M.; Madry, H., Biomaterial-guided delivery of gene vectors for targeted articular cartilage repair. *Nature reviews. Rheumatology* **2019,** *15* (1), 18--29.

12. Lolli, A.; Sivasubramaniyan, K.; Vainieri, M. L.; Oieni, J.; Kops, N.; Yayon, A.; van Osch, G., Hydrogel-based delivery of antimiR-221 enhances cartilage regeneration by endogenous cells. *Journal of controlled release : official journal of the Controlled Release Society* **2019,** *309*, 220--230.

13. Johnson, K.; Zhu, S.; Tremblay, M. S.; Payette, J. N.; Wang, J.; Bouchez, L. C.; Meeusen, S.; Althage, A.; Cho, C. Y.; Wu, X.; Schultz, P. G., A stem cell-based approach to cartilage repair. *Science (New York, N.Y.)* **2012,** *336* (6082), 717--21.

14. Maihöfer, J.; Madry, H.; Rey-Rico, A.; Venkatesan, J. K.; Goebel, L.; Schmitt, G.; Speicher-Mentges, S.; Cai, X.; Meng, W.; Zurakowski, D.; Menger, M. D.; Laschke, M. W.; Cucchiarini, M., Hydrogel-Guided, rAAV-Mediated IGF-I Overexpression Enables Long-Term Cartilage Repair and Protection against Perifocal Osteoarthritis in a Large-Animal Full-Thickness Chondral Defect Model at One Year In Vivo. *Advanced materials (Deerfield Beach, Fla.)* **2021,** *33* (16), e2008451.

15. Armiento, A. R.; Stoddart, M. J.; Alini, M.; Eglin, D., Biomaterials for articular cartilage tissue engineering: Learning from biology. *Acta biomaterialia* **2018,** *65*, 1--20.

16. Wang, Y.; Wen, F.; Yao, X.; Zeng, L.; Wu, J.; He, Q.; Li, H.; Fang, L., Hybrid Hydrogel Composed of Hyaluronic Acid, Gelatin, and Extracellular Cartilage Matrix for Perforated TM Repair. *Frontiers in bioengineering and biotechnology* **2021,** *9*, 811652.

17. Huang, B.; Li, P.; Chen, M.; Peng, L.; Luo, X.; Tian, G.; Wang, H.; Wu, L.; Tian, Q.; Li, H.; Yang, Y.; Jiang, S.; Yang, Z.; Zha, K.; Sui, X.; Liu, S.; Guo, Q., Hydrogel composite scaffolds achieve recruitment and chondrogenesis in cartilage tissue engineering applications. *Journal of nanobiotechnology* **2022,** *20* (1), 25.

18. Sun, W.; Gregory, D. A.; Tomeh, M. A.; Zhao, X., Silk Fibroin as a Functional Biomaterial for Tissue Engineering. *Int J Mol Sci* **2021,** *22* (3).

19. Shi, W.; Sun, M.; Hu, X.; Ren, B.; Cheng, J.; Li, C.; Duan, X.; Fu, X.; Zhang, J.; Chen, H.; Ao, Y., Structurally and Functionally Optimized Silk-Fibroin-Gelatin Scaffold Using 3D Printing to Repair Cartilage Injury In Vitro and In Vivo. *Advanced materials (Deerfield Beach, Fla.)* **2017,** *29* (29).

20. Guo, X.; Lin, N.; Lu, S.; Zhang, F.; Zuo, B., Preparation and Biocompatibility Characterization of Silk Fibroin 3D Scaffolds. *ACS applied bio materials* **2021,** *4* (2), 1369--1380.

21. Li, G.; Sun, S., Silk Fibroin-Based Biomaterials for Tissue Engineering Applications. *Molecules* **2022,** *27* (9).

22. Zheng, H.; Zuo, B., Functional silk fibroin hydrogels: preparation, properties and applications. *Journal of materials chemistry. B* **2021,** *9* (5), 1238--1258.

23. Zhao, Y.; Zhu, Z. S.; Guan, J.; Wu, S. J., Processing, mechanical properties and bio-applications of silk fibroin-based high-strength hydrogels. *Acta biomaterialia* **2021,** *125*, 57--71.

24. Su, D.; Yao, M.; Liu, J.; Zhong, Y.; Chen, X.; Shao, Z., Enhancing Mechanical Properties of Silk Fibroin Hydrogel through Restricting the Growth of β-Sheet Domains. *ACS applied materials & interfaces* **2017,** *9* (20), 17489--17498.

25. Wu, J.; Chen, Q.; Deng, C.; Xu, B.; Zhang, Z.; Yang, Y.; Lu, T., Exquisite design of injectable Hydrogels in Cartilage Repair. *Theranostics* **2020,** *10* (21), 9843--9864.

26. Li, J.; Chen, G.; Xu, X.; Abdou, P.; Jiang, Q.; Shi, D.; Gu, Z., Advances of injectable hydrogel-based scaffolds for cartilage regeneration. *Regenerative biomaterials* **2019,** *6* (3), 129--140.

27. Zhu, W.; Niu, T.; Wei, Z.; Yang, B.; Weng, X., Advances in Biomaterial-Mediated Gene Therapy for Articular Cartilage Repair. *Bioengineering (Basel, Switzerland)* **2022,** *9* (10).

28. Yang, R.; Chen, F.; Guo, J.; Zhou, D.; Luan, S., Recent advances in polymeric biomaterials-based gene delivery for cartilage repair. *Bioactive materials* **2020,** *5* (4), 990--1003.

29. Laysandra L, Kurniawan D, Wang CL, Chiang WH, Chiu YC. Synergistic Effect in a Graphene Quantum Dot-Enabled Luminescent Skinlike Copolymer for Long-Term pH Detection. ACS Appl Mater Interfaces. **2021**;13(50):60413-60424.

30. Kurniawan D, Mathew J, Rahardja MR, et al. Plasma-Enabled Graphene Quantum Dot Hydrogels as Smart Anticancer Drug Nanocarriers. Small. **2023**;19(20):e2206813

31. Grisolia A, De Santo M, Curcio M, et al. Engineered Mesoporous Silica-Based Nanoparticles: Characterization of Surface Properties. Materials (Basel). 2024;17(13):3352.

32. Wang W, Wang P, Tang X, et al. Facile Synthesis of Uniform Virus-like Mesoporous Silica Nanoparticles for Enhanced Cellular Internalization. ACS Cent Sci. 2017;3(8):839-846.

33. Kang H, Long DJ, Haynes CL. Preparation of Colloidally Stable Positively Charged Hollow Silica Nanoparticles: Effect of Minimizing Hydrolysis on ζ Potentials. Langmuir. 2019;35(24):7985-7994

34. Cao F, Chen Y, Wang X, et al. Therapeutic effect and potential mechanisms of intra-articular injections of miR-140-5p on early-stage osteoarthritis in rats. Int Immunopharmacol. 2021;96:107786.

35. Hwang HS, Kim HA. Chondrocyte Apoptosis in the Pathogenesis of Osteoarthritis. Int J Mol Sci. 2015 Oct 30;16(11):26035-54.

36. Chen Y, Huang H, Zhong W, Li L, Lu Y, Si HB. miR-140-5p protects cartilage progenitor/stem cells from fate changes in knee osteoarthritis. Int Immunopharmacol. 2023;114:109576.

Image deleted. Please check.

Figure 1S. The self-healing process of CaP@mSF-HA-NB.

Image deleted. Please check.

Figure 2S. The stability of the fabricated hydrogel in lysozyme/PBS solutions. The hydrogels with different concentrations of HA-NB were all degraded gradually with time in lysozyme/PBS solution. The higher the concentration of HA-NB in the hydrogel, the slower its degradation rate. And each hydrogel can be maintained for more than 2 weeks.

Image deleted. Please check.

Figure 3S. A: The particle size distribution of MON-PEI/miR-140-5p complexes in DMEM medium on day 0, day 0.5, day 1, day 3, day 7, day 14. B: The particle size distribution of MON-PEI/miR-140-5p complexes in PBS medium on day 0, day 0.5, day 1, day 3, day 7, day 14. The results show that the MON-PEI/miR-140-5p complexes remain stable.
